# In Vitro Anti-Biofilm Activities of Citral and Thymol Against *Candida tropicalis*

**DOI:** 10.3390/jof5010013

**Published:** 2019-02-03

**Authors:** Apurva Chatrath, Rashmi Gangwar, Poonam Kumari, Ramasare Prasad

**Affiliations:** Molecular Biology & Proteomics Laboratory, Department of Biotechnology, Indian Institute of Technology, Roorkee-247667 Uttarakhand, India; apurva.chatrath@gmail.com (A.C.); rashmi22gangwar@gmail.com (R.G.); bpoonam15@gmail.com (P.K.)

**Keywords:** *Candida tropicalis*, biofilm, citral, thymol

## Abstract

*Candida tropicalis* is an emerging non-*albicans Candida* species which is pathogenic to the immune-compromised humans, especially in tropical countries, including India. The acquired resistance of *Candida* species towards antifungal therapies is of major concern. Moreover, limited efficacy and dosage constraint of synthetic drugs have indicated the prerequisite of finding new and natural drugs for treatment. In the present study, we have compared the influence of citral and thymol on *C. tropicalis* and its biofilm along with expression levels of certain antifungal tolerance genes. The antifungal and anti-biofilm activities of the both were studied using 2,3-bis(2-methoxy-4-nitro-5-sulfophenyl)-2H-tetrazolium-5-carboxanilide sodium salt (XTT) reduction assay, field emission scanning electron microscope (FE-SEM) and confocal laser scanning microscope (CLSM) and real-time reverse transcription polymerase chain reaction (RT-PCR) analysis. Citral and thymol have damaged the cells with distorted surface and less viability. Quantitative real-time PCR analysis showed augmented expression of the cell membrane biosynthesis genes including *ERG11*/*CYT450* against citral and the cell wall related tolerance genes involving *CNB1* against thymol thus, depicting their differential mode of actions.

## 1. Introduction

*Candida* species are widely associated yeasts with humans in commensalism but turn into opportunistic pathogens during the extensive use of broad-spectrum antibiotics and suppression of the host immune system [1]. *Candida tropicalis* closely resembles *C. albicans* in taxonomy. Infections due to *C. tropicalis* have increased dramatically on a global scale thus, proclaiming this organism to be an emerging pathogenic yeast with higher prevalence in the Asia-Pacific and Europe regions [2,3]. *C. tropicalis* is majorly responsible for 67–90% of the epidemiological nosocomial candidaemia among non-*albicans Candida* species in India [4,5]. *C. tropicalis* is capable of proper biofilm formation resulting in frequent biofilm producing species among non-*albicans Candida* species [6,7]. Biofilm lifestyle of yeast cells represents a unique phenotypic trait of the pathogenic species under stressful conditions. This life form is vastly established as more resistant to the antifungal agents and evades immune responses of the host [8]. Abiotic surfaces such as medical devices have shown the formation of biofilm by the *Candida* species [9]. In vivo studies of rat and rabbit also depicted the similar biofilm formation on central venous catheter models [10]. Components of the immune system including neutrophils, macrophages, blood cells and platelets were found embedded in In vivo biofilm. As a consequence, researchers have now realised the fact that it is important to study the biofilm communities rather than planktonic forms for the characterization of the infectious potential of fungal pathogens.

Essential oils are well known for their antifungal properties in vitro and In vivo but its anti-biofilm activity has not been studied extensively. The major components of essential oils have well described antifungal activities [11]. Strong actions of citral and thymol have been defined in several studies with *C. albicans* and other non-*albicans Candida* species [12,13,14]. Citral and thymol are generally regarded as safe (GRAS) by the Food and Drug Administration for human consumption and food additives (Food and Drug Administration 2015a; 2015b). Citral (3,7-dimethyl-2, 6-octadienal) is the basic constituent of many plants such as lemon-grass (*Cymbopogon citratus*) and thymol (2-isopropyl-5-methylphenol) is present majorly in thyme oil (*Thymus vulgaris*) with various pharmacological as well as antimicrobial properties. Since antifungal resistance is inevitable, it is crucial to get powerful insights into the molecular mechanisms that govern the tolerance of drugs in order to hinder the resistance. An understanding of the specific targets involved in a particular agent is critical in developing novel and effective therapeutic strategies [15].

The present study investigated the comparative potentials of citral and thymol against *C. tropicalis*. The effect of citral and thymol against *C. tropicalis* and their action against *C. tropicalis* biofilm is recognized through metabolic activities and morphological changes. The relative fold change in the expression of certain key genes which are involved in major pathways followed by *C. tropicalis*, to reduce the effect of well-known drugs, has also been investigated to get the insights in the plausible mechanism for its survival in the presence of these two components.

## 2. Materials and Methods

### 2.1. Organisms, Media and Growth Conditions

The reference strain *Candida tropicalis* (NCIM-3118) used in the present study was procured from the National Chemical Laboratory (NCL), Pune, India. Studies were performed using preserved glycerol stock, regularly revived on Sabouraud dextrose agar medium (SDA, HiMedia, Maharashtra, India) at 30 °C. The cells were cultured in Sabouraud dextrose broth (SDB, HiMedia, Maharashtra, India) for 24 h at 30 °C with 200 rpm, agitation. RPMI-1640 medium with L-glutamine without sodium bicarbonate, buffered with 0.165 M morpholinepropanesulfonic acid (MOPS, HiMedia, Maharashtra, India) at pH 7 was utilized for biofilm formation. Stock solutions of amphotericin B, citral and thymol, acquired from Sigma-Aldrich, MO, USA, were freshly prepared in dimethyl sulfoxide (DMSO, CDH Fine Chemicals, India).

### 2.2. Anti-Fungal and Anti-Biofilm Susceptibility Tests

Activities of citral and thymol against planktonic cells were tested by broth microdilution method using Clinical and Laboratory Standards Institute (CLSI) document M27, guidelines [16]. Planktonic cells were grown in SDB for 24 h at 30 °C with 200 rpm. The harvested cells were washed using sterile phosphate buffered saline (1 × PBS, 0.1 M, pH 7.4) and resuspended at 2 × 10^3^ cells/mL concentration in the RPMI-1640 medium. Serially double diluted concentrations from 0 µg/mL to 1024 µg/mL of citral and thymol were prepared in RPMI-1640, respectively and the cell suspension of 100 µL was added to each well of 96-well microtiter plates so that the final working volume contains 1 × 10^3^ cells/mL with the final DMSO concentration not exceeding more than 5% in any assay. Pre-sterile 96-well polystyrene microtiter plates (Tarsons, West Bengal, India) were set with 100 µL of each dilution as treatment and control well contained 5% DMSO dispensed in RPMI-1640. The plates were then incubated at 37 °C for 48 h and growth was measured using 96-well plate reader (SpectraMax, Molecular Devices, CA, USA), in terms of optical density (OD) at 600 nm [17]. The biofilm formation assay was performed in 96-well polystyrene microtiter plates as described earlier [18,19]. Briefly, the cells at a concentration of 2 × 10^6^ cells/mL were suspended in a 100 µL volume in each well in RPMI-1640. Serial double dilutions of citral and thymol were made in RPMI-1640 and added to each well as treatment while acquiring a final concentration of cells as 1 × 10^6^ cells/mL and concentrations of citral and thymol ranging from 0 µg/mL to 1024 µg/mL for each. Control wells were prepared with 5% DMSO in RPMI-1640. Plates were incubated at 37 °C for 48 h. For preformed biofilms, the cells were suspended at a concentration of 1 × 10^6^ cells/mL, taking 100 µL of suspension in each well of 96-well polystyrene plates. The plates were then, incubated at 37 °C for 24 h. After incubation, non-adherent cells were removed through aspiration of media and the wells were washed thrice using 1 × PBS. Residual PBS was removed by inverting the plates over blotting sheets. A volume of 100 µL of each serially double diluted concentration (0 µg/mL to 1024 µg/mL) of citral and thymol, not exceeding DMSO by 5%, was added to each well with treatment, whereas, control wells were made using 5% DMSO in RPMI-1640. The plates were incubated at 37 °C for another 24 h. The metabolic activity of biofilm was quantitatively determined by colourimetric XTT [2,3-bis(2-methoxy-4-nitro-5-sulfophenyl)-2H-tetrazolium- 5-carboxanilide sodium salt] reduction assay.

After appropriate incubations of microtiter plates, media was aspirated and biofilm was washed using 1 × PBS. Colourimetric XTT reduction assay was performed as reported earlier [19]. A stock solution of 5 g/L XTT tetrazolium salt (Sigma-Aldrich, MO, USA) was mixed in 10 mL of PBS to get 0.5 g/L working solution. The 1 µM final concentration of menadione (Sigma-Aldrich, MO, USA) was added to XTT. The working XTT-menadione solution of 100 µL was dispensed into the wells containing prewashed biofilm and to the control wells (for background subtraction). All the plates were incubated at 37 °C in the dark for 1 h and absorbance were read at 492 nm. Colour change due to formazan formation was directly correlated to the metabolic activity. Percentage killing of each component was calculated using the formula [1 − (OD_492_ sample / OD_492_ control)] × 100%.

The MIC_50_, BIC_50_ and BEC_50_ (minimum inhibitory concentration of planktonic cells, biofilm inhibitory concentration and biofilm eradicating concentration, respectively at which the cell growth was reduced to 50% to that of the healthy cells) for *C. tropicalis* were determined using microdilution method (as described above) in the treated/untreated wells from 0 µg/mL to 1024 µg/mL supplemented with 400 µg/mL of ergosterol and 0.8 M sorbitol as an osmotic supporter, obtained from Sigma-Aldrich, MO USA to study the ergosterol binding and sorbitol effect, respectively [20]. Amphotericin B (1 µg/mL, 4 µg/mL and 8 µg/mL) was taken as the control drug for the ergosterol assay.

### 2.3. Field Emission Scanning Electron Microscope (FE-SEM) and Confocal Laser Scanning Microscope (CLSM)

Qualitative analysis of the effect of citral and thymol on biofilm was evaluated by field emission scanning electron microscope (FE-SEM) (Carl Zeiss AG, EVO 40, Oberkochen, Germany) and confocal laser scanning microscope (CLSM), LSM -700 (Carl Zeiss, Oberkochen, Germany) as described in the earlier study [21], with slight modifications. Biofilm was formed on silicone elastomer discs in 12-well cell culture plates (Tarsons, West Bengal, India). Catheters were disinfected and treated with sterile foetal bovine serum (FBS) (Gibco, Thermo Fisher Scientific, MA, USA). Briefly, FBS treated catheters were dispersed with a cell concentration of 1 × 10^6^ cells/mL in RPMI-1640 and were incubated at 37 °C for 24 h. The biofilm formed on the elastomer surfaces was washed using 1 × PBS and incubated for another 24 h in the presence of amphotericin B (8 µg/mL) BEC_50_ concentration that is, 128 µg/mL of both components. Control biofilm was formed in the presence of 5% DMSO in RPMI-1640. At the end of biofilm formation, for FE-SEM, biofilm was dried and processed further. Briefly, biofilm was washed using PBS and were subsequently fixed in 2% (*v/v*) glutaraldehyde (HiMedia, Maharashtra, India), followed by dehydration in a series of 25%, 50%, 75% and 100% of the ethanol (Merck, MA, USA). Finally, the elastomer discs were glued on stubs and sputter coated with gold for 30 s. The biofilm was then examined using FE-SEM.

For CLSM, biofilm was formed on silicone elastomer in the presence of the BEC_50_ (128 µg/mL) concentration of citral and thymol; and control biofilm was formed in RPMI-1640 with 5% DMSO. The biofilm was then, stained with fluorescent stains FUN-1 (Molecular Probes, Invitrogen, MA, USA) at working concentration of 1 mM and Concanavalin-Alexa Fluor 488 conjugate (Invitrogen, MA, USA) at working concentration of 0.5 g/L following incubation for 0.5 h at 37 °C in dark, the biofilm was then observed using ZEN imaging software and analysed using Image J 1.8.0 software. The images were obtained in series to get the three dimensional view, each positioned at the intervals of 1 µm in *z*-axis.

The three-dimensional biofilm structures were quantified using COMSTAT software [22]. The stacks of images which were obtained using CLSM were further analysed by exporting these z-series images to COMSTAT plugin of Image J software. Biomasses, average thickness, surface to bio-volume ratio and substratum coverage were considered parameters to be analyzed.

### 2.4. Measurement of Reactive Oxygen Species (ROS) Levels

The intracellular ROS levels in *C. tropicalis* biofilm were assessed using the fluorescent dye chloromethyl-dicholodihydrofluorescein diacetate (CM-H2DCFDA) from Sigma-Aldrich, MO, USA in the presence of both at BIC_50_ (64 µg/mL for citral and 32 µg/mL for thymol) and BEC_50_ (128 µg/mL of each) [23]. Amphotericin B (BIC_50_: 4 µg/mL and BEC_50_: 8 µg/mL) was taken as a control drug. Briefly, the biofilm cells with/without treatment were added with 20 µM CM-H2DCFDA and incubated at 37 °C for 1 h. The fluorescence intensity was measured with excitation (485 nm) and emission (535 nm) using fluorescent microplate reader (BioTek, Synergy H1 Hybrid Multi-mode Microplate Reader, VT, USA).

### 2.5. RNA Isolation, cDNA Synthesis and Real-Time Expression Analysis

The RNA was isolated from the control and treated biofilm cells at BEC_50_ concentrations of citral and thymol that is, 128 µg/mL of each which were grown for 48 h using RNA-XPress reagent (HiMedia, Maharashtra, India) according to the manufacturer’s protocol. Briefly, ~200 mg of the cells with/without treatment were crushed in liquid nitrogen and treated with the reagent. The obtained gel-like pellet was finally dissolved in RNase-free water and was quantified using NanoDrop 1c (Thermo Scientific, MA, USA). Genomic DNA contamination was removed by RNase-free–DNase Set (Qiagen, Hilden, Germany). Reverse transcription was performed in a total volume of 20 µL using 2 µg of RNA by first strand cDNA synthesis kit (Thermo Scientific, MA, USA), following prescribed protocol. To evaluate the gene expression levels of key genes responsible for antifungal resistance at the transcriptomic level, real-time RT-PCR was performed. The primers (GCC Biotech, West Bengal, India) used for this study are listed in Table 1.

The normalisation was done using glyceraldehyde-3-phosphate dehydrogenase (*GAPDH*). RT-PCR was carried out using SYBR Green PCR-Master Mix (Roche, Basel, Switzerland) in replicates with three sets of experiments. PCR mix (10 µL SYBR Green, 1.25 µL cDNA, 2 µL primer mix and 6.75 µL ddH_2_O) was used for each gene. The RT-PCR was performed (Step 1: 95 °C for 3 min, Step 2: 95 °C for 15 s, 51.5 °C for 45 s and 72 °C for 30 s for 40 cycles) using Eppendorf Realplex Master Cycler. The data was analysed to determine the fold change in the expression of the gene by calculating ratio = 2^−ΔΔC_t_^, using C_t_ values [24].

### 2.6. Statistical Analysis

All of the experiments were carried out in triplicates and were expressed as mean values with the corresponding standard deviations (SD). Statistical significance between treated and control groups was analysed through one-way analysis of variance (ANOVA) with the *p*-value < 0.05 by using SigmaPlot 14.0 software.

## 3. Results and Discussion

### 3.1. Citral and Thymol Effectively Inhibited C. tropicalis and Eradicated Biofilm

The *C. tropicalis* planktonic cells and its biofilm were grown in serially double diluted concentrations (0–1024 µg/mL) of the citral and thymol and the minimum inhibitory concentration values (MICs) were determined as shown in Table 2. Interestingly, it was observed that citral (MIC_50_ = 32 µg/mL) and thymol (MIC_50_ = 16 µg/mL) were significantly effective against planktonic cells of *C. tropicalis* [25]. The anti-biofilm activity against *C. tropicalis* was also tested in vitro and their effects were studied through XTT reduction method. To calculate the inhibitory effect on the biofilm formation of *C. tropicalis*, both citral and thymol were added at the initial stage of biofilm formation. The process of biofilm formation includes initial adherence of the planktonic cells to the abiotic surfaces, followed by micro-colony formation and release of extracellular matrix which is due to increase in the metabolic activity of *Candida* species during proliferation and finally stable growth at maturation [26]. For preformed biofilm, the components were added after 24 h (log phase) of biofilm formation. The BIC_50_ value for citral was determined to be 64 µg/mL while thymol inhibited biofilm formation at a lower concentration of 32 µg/mL.

The mature biofilm is a complex structure acting as the reservoir of cells under stressful conditions and is responsible for the dispersal of cells during infection. The BEC_50_ for both citral and thymol were observed to be 128 µg/mL as the effective concentrations to eradicate matured *C. tropicalis* biofilm. It was outlined that two and four fold higher concentrations are needed to eradicate mature biofilm than to inhibit its formation in the case of citral and thymol, respectively. Also, thymol is more effective in the inhibition of biofilm formation and becomes comparatively less effective on preformed biofilm due to the recalcitrant nature of the mature biofilm. Furthermore, the extracellular matrix limits the penetration of antimicrobial agents into the biofilm as suggested by the observed inhibitory concentrations of citral and thymol during biofilm formation. This could be partly because of diffusion limitation affected by the 3-dimensional structure but primarily due to absorption or reaction of the antimicrobial agent within extracellular matrix components. The results suggested that the absorption and consumption of thymol was more in comparison to citral [27]. This may occur in the surrounding part of the biofilm and thus, neutralizes the more reactive antifungal agent such as thymol in this case. A previous report with azole group of standard drugs such as fluconazole has demonstrated that the BEC were eight fold higher than BIC [28]. Since citral and thymol have shown a good effect against planktonic cells and biofilm forms of *C. tropicalis*, further, the morphological changes in the biofilm form were examined.

### 3.2. FE-SEM and CLSM Analysis Displayed Damage to The Biofilm in The Presence of Citral and Thymol

**Figure 1:**
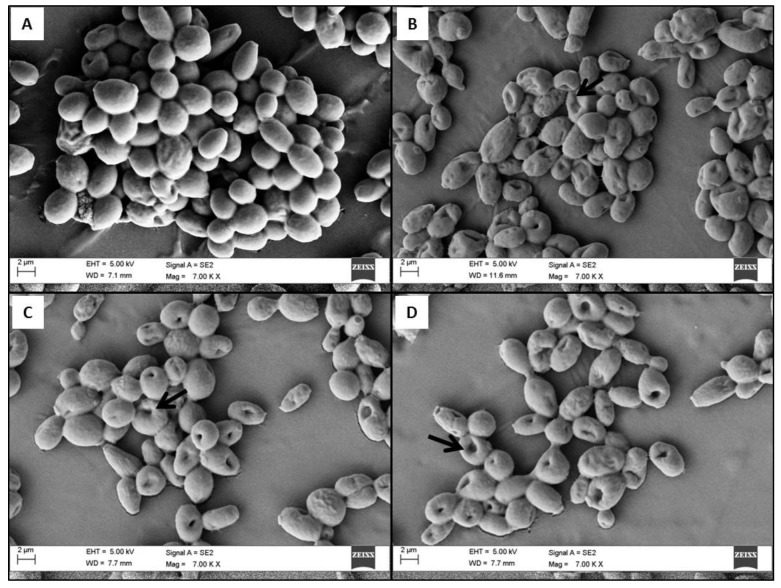
Images through field emission scanning electron microscopy (FE-SEM) (Magnification: 7000 X, Bar: 2 µm) of *C. tropicalis* biofilm; (**A**) control (1% DMSO) with densely clustered healthy cells; (**B**) amphotericin B (1 µg/mL); (**C**) citral (128 µg/mL); and (**D**) thymol (128 µg/mL), cells are more porous and flaccid with loosen cell surface. # Black arrows show pores in the cells.

The morphological changes of preformed *C. tropicalis* biofilm and their cellular surfaces in the presence of BEC_50_ (128 µg/mL) of citral and thymol were investigated. The morphologies of the biofilm with and without treatment were observed when proceeded with the visualization of mature biofilms through FE-SEM and CLSM analysis. During FE-SEM imaging, control (0 µg/mL) biofilm displayed complex structure with clustered cells surrounded by the extracellular matrix as shown in Figure 1A. However, during the treatment of amphotericin B (1 µg/mL); BEC_50_ (128 µg/mL) of citral and thymol, the biofilm exhibited deformed morphology with cells having porous outer membrane as represented in Figure 1B–D, respectively. Additionally, the deformation of the cells and loss of cell membrane integrity have been reported as the mechanisms of antimicrobial activity [29]. These morphological alterations of the cells could be associated with the loss of cell membrane integrity ultimately resulting in cell death [30].

**Figure 2:**
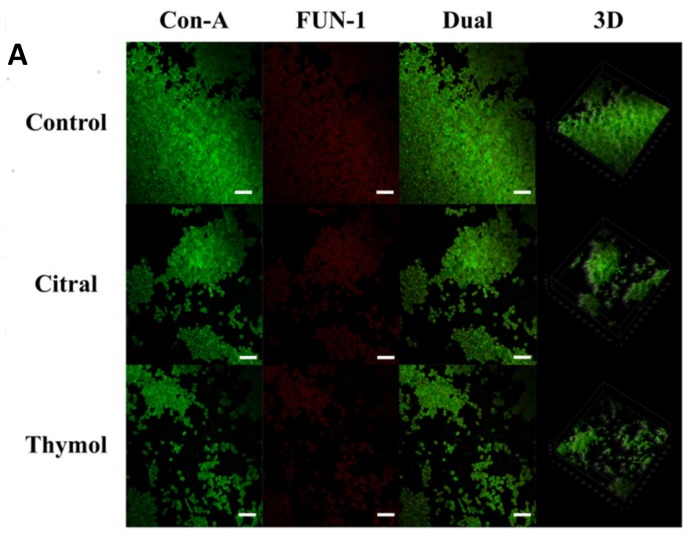

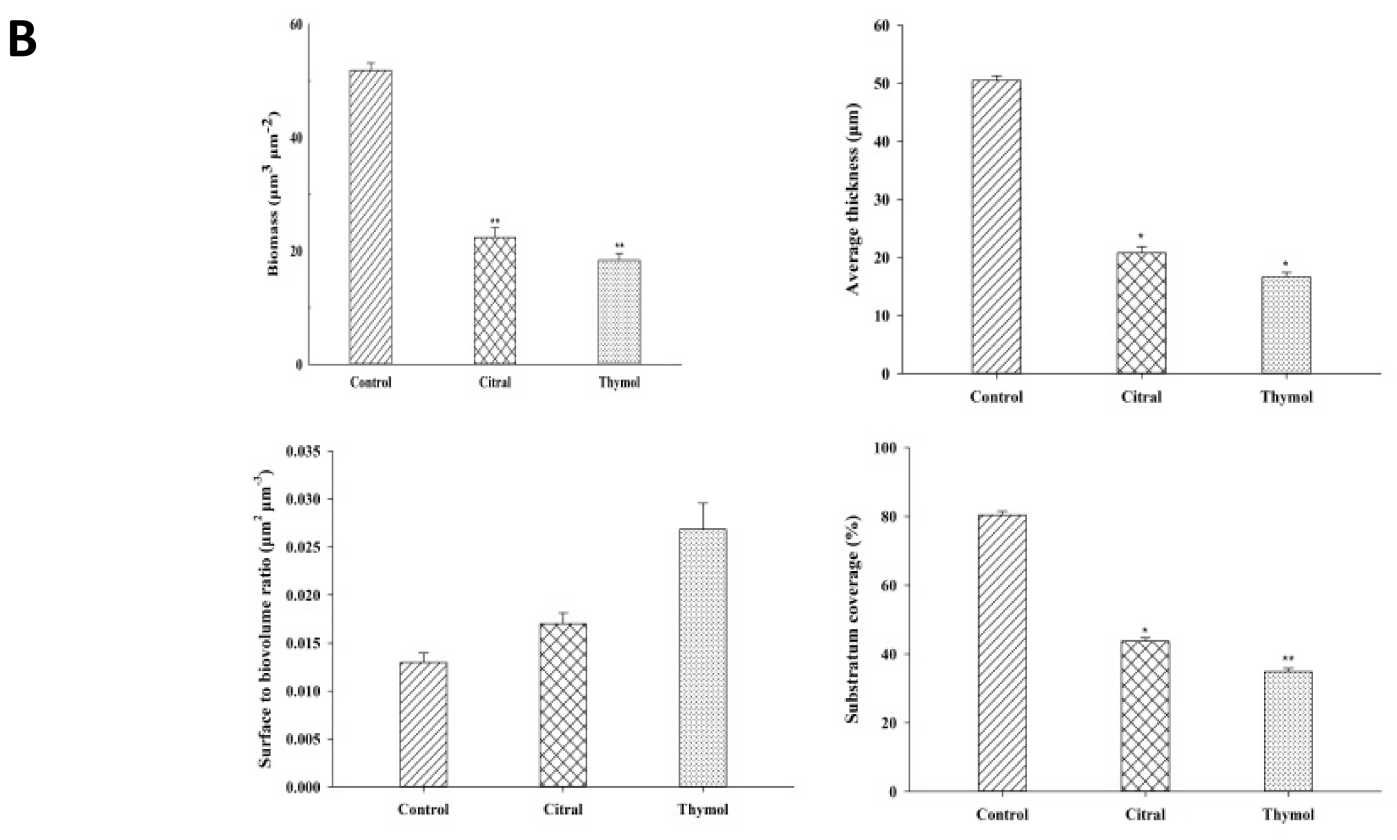
(**A**) Confocal laser scanning microscopic images (CLSM) of *C. tropicalis* biofilm; dual stained: CON-A (Excitation: 488, Emission: 505), staining polysaccharide walls, green and FUN-1 (Excitation: 470, Emission: 590), staining metabolically active cells, red; thus dually staining yellowish-green colour of healthy biofilm; control (1% DMSO). Power field: 40×; Scale Bar: 50 µm; (**B**) COMSTAT analysis of *C. tropicalis* biofilm.

During CLSM analysis, the combination of fluorescent dyes Con-A (selectively binds mannose and glucose residues of cell wall polysaccharides) and FUN-1 (cytoplasmic probe for cell viability) was used. Intense green fluorescence resulting from Con-A binding to cell wall polysaccharides outlined the cell walls of the yeast, whereas red colour appeared due to FUN-1 staining localized in dense aggregates in the cytoplasm of metabolically active cells [31]. Thus, areas of red fluorescence represent metabolically active cells and green fluorescence indicated cell wall-like polysaccharides, whereas yellow areas represent dual staining [32]. CLSM images of control biofilm revealed dense and compact structure which was green with Con-A staining cell wall and red with FUN-1 staining live cells, conversely, with yellowish-green in colour in the presence of both Con-A and FUN-1 that is, dual stain. However, when treated with citral and thymol at BEC_50_ that is, 128 µg/mL, reduction in dense live cells and more green cells/broken cell walls as debris was observed as in Figure 2A.

Furthermore, COMSTAT analysis depicted the lower biomasses in the presence of both citral and thymol, less thickened biofilm at matured stage and lesser substratum coverage when compared to the healthy biofilm. Also, the surface to bio-volume ratio was increased showing less biofilm formation during treatment as represented in Figure 2B.

### 3.3. Citral and Thymol Indicated No Direct Binding to The Cell Membrane but Thymol Acts via Cell Wall

The binding of citral and thymol to the fungal membrane sterol was determined through the change in inhibitory concentration values (ICs) of both agents for *C. tropicalis* in the presence and absence of ergosterol. The exogenous ergosterol would prevent the binding to the fungal membrane’s ergosterol if the activity of citral or thymol was instigated by binding to ergosterol. The ICs enhancement when substituted with the exogenous ergosterol with respect to the control assay was witnessed with no altercations in MIC_50_, BIC_50_ and BEC_50_ as shown in Table 2. Recent studies have revealed that citral inhibits ergosterol biosynthesis in *C. albicans* and *Penicillium italicum* but does not binds directly to the ergosterol. In accordance with the previous reports, both citral and thymol do not bind to the cell membrane of *C. tropicalis* [33,34]. However, a four-fold increase of ICs was observed with positive control drug amphotericin B, whose interaction with ergosterol is well established.

An osmotic stabilizer such as sorbitol in the media could reverse the antifungal effect of the agents which targets the fungal cell wall. When the *C. tropicalis* was treated with both citral and thymol in the media supplemented with sorbitol, the IC values were not affected in the presence of citral. However, in the case of thymol, the shift of MIC_50_ from 16 µg/mL to 32 µg/mL and BIC_50_ from 32 µg/mL to 64 µg/mL suggested that thymol would act as an inhibitor of the cell wall synthesis or assembly.

### 3.4. Thymol and Citral Generated Reactive Oxygen Species

**Figure 3:**
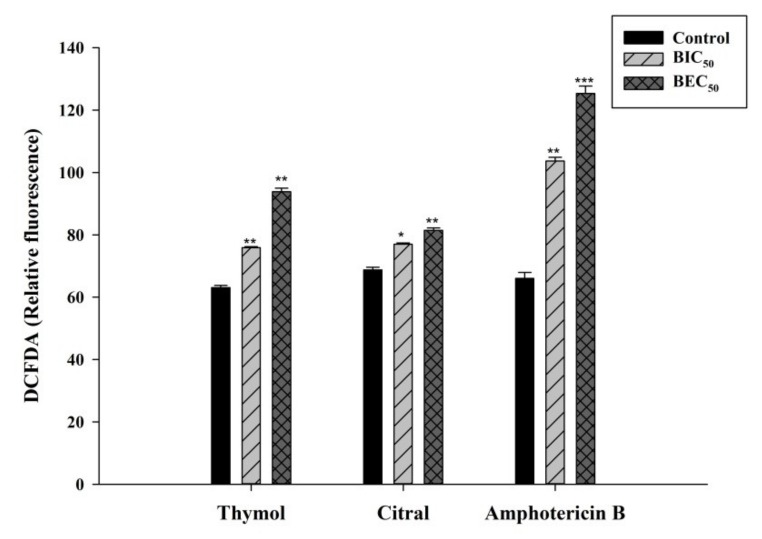
Graphical representation of the DCFDA fluorescence of *C. tropicalis* biofilm representing ROS production during treatment of amphotericin B, citral and thymol. Error bars denote standard deviation (SD).* *p* < 0.05, ** *p* < 0.01, ^***^ *p* < 0.005 when compared with the control.

Mono-terpene phenols such as thymol irrationally increase the production of reactive oxygen species (ROS) in *C. albicans*, which negatively affects the antioxidant system [35]. ROS production due to oxidative stress leads to DNA damage and defective repair machinery [36]. Here, thymol has shown significant increment in the ROS production than citral as represented in Figure 3.

### 3.5. Citral Upregulated ERG11/CYT450 Genes Whereas Thymol Upregulated CNB1 and SOD1 Genes

**Figure 4:**
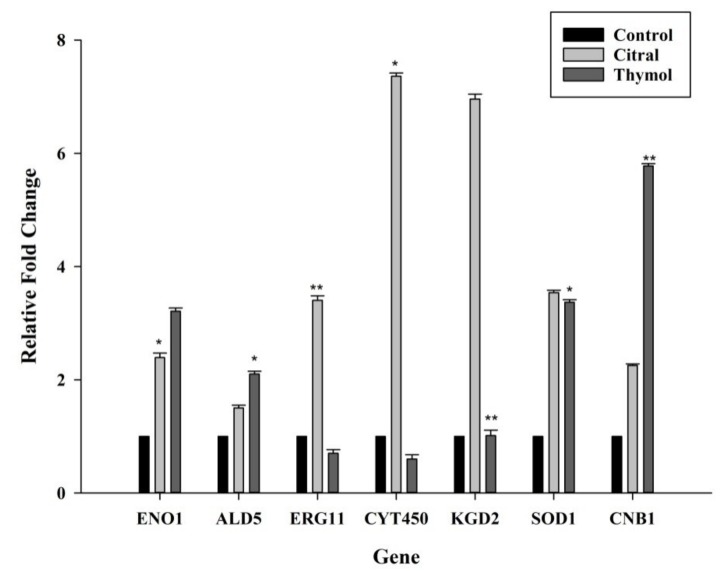
Effect of citral and thymol on the relative expression of the selected genes in *C. tropicalis*. Error bars represent standard deviation (SD). * *p* < 0.05, ** *p* < 0.01 in comparison to the control.

The relative gene expression of *ENO1*, *ALD5*, *ERG11*, *CYT450*, *KGD2*, *SOD1* and *CNB1*, responsible for major antifungal tolerance mechanism were evaluated as depicted in Figure 4. The expression of *ENO1* (enolase), involved in virulence and drug resistance, was increased when treated with citral and thymol. The null mutants have demonstrated various degrees of increased antifungal drug susceptibility compared to SC5314 strain, reporting the role of *ENO1* in drug resistance mechanism [37].

The *ALD5* (alcohol dehydrogenase) gene was overexpressed in the case of both citral and thymol, indicating its role in the tolerance mechanism. *ALD5*, is a redox-related gene involved in oxidative stress defence. The overexpression of *ALD5* verifies the activation of the anti-oxidative system to restore the redox balance during antifungal activity of these agents as in agreement with the previous study of drug resistance [38].

*CYT450* (cytochrome P-450) dependent enzyme *ERG11* (lanosterol 14α-demethylase) is a specific target to azole resistance and was checked for their involvement in the tolerance of citral and thymol as antifungals. The increment in the expression of the *ERG11* and *CYT450* was observed with the treatment of citral but was not observed in the presence of thymol. Also, *CYT450*, an azole binding protein has shown augmented expression than *ERG11*. The upregulation of *ERG11* has only a minor effect on drug tolerance and does not exceed a factor of three or five. Whereas, upregulation of *CYT450* in the presence of citral corresponds to the activation of drug tolerance mechanism similar to that of fluconazole [39]. Thus, the increased expression of *ERG11* and *CYT450* in the presence of citral explains the role in the assembly of the cell membrane as mentioned in the ergosterol assay where no direct attachment to the membrane was perceived.

*KGD2* encodes the dihydrolipoyllysine-residue succinyltransferase component of 2-oxoglutarate dehydrogenase complex serves as a novel immunogenic protein that could be associated with the pathogenesis of *C. tropicalis* [40]. Expression of *KGD2* was increased in the presence of citral but was not affected when treated with thymol.

The expression of *SOD1* (copper-zinc superoxide dismutase) was significantly augmented in the presence of both citral and thymol, respectively. *SOD1* mitigates the generation of ROS by quenching fatal superoxides restoring the redox balance essential for cell survival. Antifungal drugs increase oxidative stress which induces adaptive response resulting in an increased expression of *SOD1* [41].

*CNB1* (calcineurin regulatory subunit) which is required for cell wall integrity and drug tolerance has also shown increased expression in the presence of citral as well as thymol. *C. tropicalis* calcineurin mutants have shown susceptibility to the antifungal agents targeting the cell wall and serve as a negative regulator of calcium tolerance function. Increased expression of *CNB1* in the presence of thymol indicates its plausible role in cell wall synthesis or assembly, which corroborated the sorbitol assay.

The undergone study has documented that both citral and thymol showed moderate anti-myocotic activity against *C. tropicalis*. Thymol was found to be more effective against *C. tropicalis* planktonic cells and biofilm inhibition than citral. However, during the eradication of the biofilm, thymol represented relatively low and equivalent activity to that of citral due to extra impervious nature of the mature biofilm. Interestingly, it was observed that citral as an antifungal agent, targets the cell membrane whereas thymol aims the cell wall. These were evident by the differential expression of *ERG11/CYT450* and *CNB1* and sorbitol protection assay against citral and thymol. Exogenous ergosterol binding assay directed that the citral does not bind directly to the ergosterol. Although both, citral and thymol exert similar antifungal activity yet different action on cell wall and membrane depicts their diverse mode of action. This finding suggests imminent pharmacological applications for developing alternative treatments against opportunistic mycoses. It is also advised to study the varying pathways involved in the survival of cells upon treating with different antifungal agents to elaborate the targets of each for further drug-delivery modulations for actual manifestations.

## Figures and Tables

**Table 1:** Primers used for real-time RT-PCR.

Gene	Gene ID	Amplicon Length (bp)	T_m_ (°C)	Sequence 5’ to 3’
enolase	*ENO1*	176	50.2	F: TATTGCCATGGATGTTGCTT
enolase	*ENO1*			R: CTTCAGCGAATGGATCTTCA
alcohol dehydrogenase	*ALD5*	196	51.8	F: TTGTTACCGGTGGTGCTAGA
alcohol dehydrogenase	*ALD5*			R: GAGTGAATACCAGCAGCCAA
sterol 14-demethylase	*ERG11*	120	50.5	F: ACTCATGGGGTTGCCAATGT
sterol 14-demethylase	*ERG11*			R: AGTTGAGCAAATGAACGGTC
Cytochrome P450 52A1	*CYT450*	169	54.0	F: GTTCTGCTGTGTTTCCAGCC
Cytochrome P450 52A1	*CYT450*			R:AGACCCAGAGAATGTCAAGGC
2-oxoglutarate dehydrogenase complex	*KGD2*	135	55.0	F: GGTGCATTCTCCAAGGCTGT
2-oxoglutarate dehydrogenase complex	*KGD2*			R: CAAACCCTTTGGTGTGGCAA
superoxide dismutase	*SOD1*	140	54.0	F: TTCAAGGTTCTGGTTGGGCT
superoxide dismutase	*SOD1*			R: AGCATGTTCCCAAGCATCAA
calcineurin subunit B	*CNB1*	112	55.0	F: AGATGGGTCAGGGGAAATTGAC
calcineurin subunit B	*CNB1*			R: ACGACCATCACCATCTGTGTC
glyceraldehyde-3-phosphate dehydrogenase	*GAPDH*	153	54.0	F: GTCAACGATCCATTCATTGC
glyceraldehyde-3-phosphate dehydrogenase	*GAPDH*			R: AGCTGGGTCTCTTTCTTGGA

**Table 2:** IC values (µg/mL) of drugs in the absence and presence of sorbitol (0.8 M) and ergosterol (400 µg/mL) against *C. tropicalis*.

Drug		Sorbitol			Ergosterol	
		Control	Absence	Presence	Absence	Presence
**Citral**	^#^MIC_50_	+	32	32	32	32
	^#^BIC_50_	+	64	64	64	64
	^#^BEC_50_	+	128	128	128	128
**Thymol**	^#^MIC_50_	+	16	32	16	16
	^#^BIC_50_	+	32	64	32	32
	^#^BEC_50_	+	128	128	128	128
**Amphotericin B**	^#^MIC_50_	+	NA	NA	1	16
	^#^BIC_50_	+	NA	NA	4	32
	^#^BEC_50_	+	NA	NA	8	64

#Values represent the arithmetic means (*p* < 0.05) of the effective concentration against planktonic cells and biofilms. Amphotericin B (positive control); NA: not tested in the sorbitol assay, +: presence of the fungal growth in medium plus sorbitol and no drug.
